# Molecular Mechanism of *Cinnamomum cassia* against Gastric Damage and Identification of Active Compounds

**DOI:** 10.3390/biom12040525

**Published:** 2022-03-30

**Authors:** Myong Jin Lee, Hye Jin Seo, Gwi Seo Hwang, Sungyoul Choi, Shin Jung Park, Sung-Joo Hwang, Ki Sung Kang

**Affiliations:** 1 College of Korean Medicine, Gachon University, Seongnam 13120, Korea; myongene@naver.com (M.J.L.); seoul@gachon.ac.kr (G.S.H.); pc1075@gachon.ac.kr (S.C.); 2 Yonsei Institute of Pharmaceutical Sciences & College of Pharmacy, Yonsei University, 85 Songdogwahak-ro, Yeonsu-gu, Incheon 21983, Korea; hyejins@ckdpharm.com; 3 Chong Kun Dang (CKD) Pharm Research Institute, Yongin-si 16995, Korea; parksj@ckdpharm.com

**Keywords:** *Cinnamomum* *cassia* extract (ECC), 4-hydroxycinnamaldehyde, RAW 264.7, inflammation

## Abstract

*Cinnamomum* *cassia* is a natural product found in plants that has been used as a folk remedy for inflammation. In this study, we investigated the mechanism underlying the anti-inflammatory and antioxidant properties of *C.* *cassia* extract (ECC) in lipopolysaccharide (LPS)-induced murine RAW 264.7 cells, in comparison with 4-hydroxycinnamaldehyde, a *C. cassia* extract component. ECC and 4-hydroxycinnamaldehyde inhibited the production of nitrite oxide in a dose-dependent manner and did not show any change in cellular toxicity when treated with the same dose as that used in the nitrite assay. Moreover, they attenuated ROS accumulation after lipopolysaccharide (LPS) stimulation. ECC and 4-hydroxycinnamaldehyde decreased the mRNA and protein expression levels of inflammatory mediators (iNOS and COX-2) and cytokines such as TNF and IL-6. We also found that ECC and 4-hydroxycinnamaldehyde mitigated the phosphorylation of ERK, JNK, and transcription factors, such as NF-κB and STAT3, suppressing NF-κB nuclear translocation in LPS-activated macrophages. In addition, administration of ECC in a Sprague Dawley rat model of acute gastric injury caused by indomethacin significantly increased the gastric mucus volume. Analysis of serum and tissue levels of inflammatory mediators revealed a significant decrease in serum PGE2 and myeloperoxidase levels and a reduction in gastric iNOS, COX-2, and p65 protein levels. Collectively, these results suggest that ECC has antioxidant and anti-inflammatory effects and is a potential candidate for curing gastritis.

## 1. Introduction

The prevalence of gastritis in South Korea is rapidly escalating owing to an increase in *Helicobacter pylori* infection and fast-eating habits. Gastritis occurs when the lining of the stomach becomes inflamed; this inflammation weakens the gastrointestinal mucosa, and damage to the mucosa membrane progresses [1]. As part of the body’s defense mechanism, inflammation is a complex biological response accompanied by the activation of many types of immune cells, including macrophages, neutrophils, and lymphocytes. In particular, macrophages play critical roles in the immune response, allergy, and inflammation and protect the body from external pathogens through phagocytosis [2]. During this process, macrophages produce various inflammatory mediators such as interleukin (IL)-1β, tumor necrosis factor α (TNF-α), nitric oxide (NO), and prostaglandins [3]. Lipopolysaccharide (LPS) is a staple endotoxin that is made up of the outer membrane of Gram-negative bacteria and plays a leading role in the inflammatory reaction [4]. For example, it activates nitric oxide (NO), TNF-α, cyclooxygenase-2 (COX-2), and IL-6 in murine macrophages during the inflammatory response [5].

Nuclear factor (NF-κB), a multipotent transcription factor, is associated with inflammation and immune responses and is induced by a range of stimuli. Upon activation of the catalytic subunit of NF-κB, the inhibitor of nuclear factor kappa B (IκB) is phosphorylated by IkB kinase (IKK) and eventually leads to proteasomal degradation. Free RelA (p65) translocates to the nucleus and activates the transcription of target genes such as pro-inflammatory mediators and cytokines including iNOS, COX-2, nitric oxide, TNF-α, and IL-1β [6]. Mitogen-activated protein kinases (MAPKs) are one type of serine/threonine protein kinases that are involved in cellular processes, such as stress responses, proliferation, differentiation, and immune defense. The extracellular signal-regulated kinase (ERK), c-Jun N-terminal kinase (JNK), and p38 pathways belong to three well-known families of MAPK signaling [7]. They are activated in several macrophages under various stimuli [8]. Prostaglandins, NO, and inducible NO synthase (NOS), as well as a wide array of inflammatory mediators, such as TNF, IL-1β, and IL-6, are generally controlled by MAPKs [7]. Therefore, suppression of the NF-κB and MAPK signaling pathways can be a good solution for treating some inflammatory diseases, and the pathways can act as core targets in the inflammatory process of the gastrointestinal mucosa.

Plant-derived phytochemicals have emerged as novel agents for the treatment of gastritis. Nonsteroidal anti-inflammatory drugs (NSAIDs) are the most commonly used anti-inflammatory drugs for pain relief. However, NSAIDs have been reported to cause gastritis along with ulceration and bleeding through apoptosis and inflammation. In this study, we explored the potential pharmacological activities, including efficacy and mechanisms of action, of *Cinnamomum cassia* against gastritis. *C. cassia* is used in traditional medicine worldwide and is known to have antioxidant, anti-inflammatory, and antimicrobial effects. In addition, it is related to cardiovascular disease, common cold, and gynecological and chronic gastrointestinal disorders [9]. Its extracts contain several active components such as cinnamic aldehyde, cinnamic alcohol, cinnamic acid, and coumarin [10]. Several studies have reported only the protective effects of natural products with no side effects and toxicity against gastric mucosal damage using animal models [1,11]. Additionally, only a few studies have investigated *C. cassia*’s anti-inflammatory effects. Further, research and efforts to develop a reliable “standardization of natural drugs” by establishing the best-quality evaluation system are limited.

Therefore, we evaluated the anti-inflammatory activity of a standardized extract of *C. cassia* (ECC), compared it with that of *Artemisia* extract (AE) and lansoprazole as a positive control, and further explored its mechanism of action in the LPS-induced inflammatory response of RAW 264.7 macrophages. Moreover, an animal study was conducted to evaluate the effect of ECC administration on acute gastric injury caused by indomethacin in Sprague Dawley rats.

## 2. Materials and Methods

### 2.1. Plant Material and Chemicals

*C. cassia* (*Lauraceae*) bark was purchased from YingBai, Vietnam, and voucher specimens were obtained from the Chong Kun Dang Research Institute, Korea. The ECC was manufactured by Chong Kun Dang (Yongin, Korea). Briefly, *C. cassia* (100 g) was extracted in distilled water (800 mL) using a heat reflux extractor for 5 h. The extract was concentrated using reduced pressure in the evaporator and dry powdered (5 g). The ECC was analyzed using an Alliance HPLC system (Waters e2695, Milford, MA, USA). The result was detected under UV irradiance at 305 nm using a Phenomenex Gemini column (4.6 × 250 nm, 5 μm), and 10 μL of the sample was injected with a flow rate of 1.0 mL/min. The standard reagents used were coumarin and cinnamic acid (Sigma-Aldrich, St. Louis, MO, USA).

### 2.2. Cell Viability Assay

RAW 264.7 cells, a murine macrophage cell line, were cultured in DMEM supplemented with 10% FBS, penicillin G (100 U/mL), and streptomycin (100 μg/mL) at 37 °C in a 5% CO2 humidified atmosphere.

Cell viability was measured using an EZ-cytox assay kit (DoGenBio, Seoul, Korea). The cells (5 × 10^4^ cells/well) were seeded in a 96-well plate. The cells were treated with various concentrations (200, 100, 50, and 25 µg/mL, or 50, 25, 12.5, and 6.5 µM, respectively) of ECC and its components for 24 h. EZ-cytox reagent was added to the plate and incubated for 1 h at 37 °C. The optical density was measured at 540 nm using a plate reader.

### 2.3. Nitrite Assay

Macrophage cells were seeded in a 96-well plate at a density of 5 × 10^4^ cells/well. The cells were pretreated at the same concentration as that for cell viability. After 2 h, the cells were treated with 100 ng/mL LPS for 22 h. Each supernatant was transferred to a new 96-well plate and mixed with an equal volume of Griess reagent. The absorbance was measured at 550 nm using a microplate reader.

### 2.4. Reverse Transcription Quantitative Polymerase Chain Reaction (RT-PCR)

Total RNA was extracted using the RNeasy Mini Kit following the manufacturer’s instructions (Qiagen Inc., Valencia, CA, USA). Total RNA (1 µg) was reverse transcribed into complementary DNA using a commercially available cDNA synthesis kit (Revertaid First Strand cDNA; Thermo Scientific, Eugene, OR, USA).

Quantitative RT-PCR was performed using the Accupower 2xGreenstar qPCR master mix (Bioneer, Daejeon, Korea) with a Quantstudio 3 real-time PCR system (Applied Biosystems, Waltham, CA, USA). The mRNA expression level was calculated using the 2_−∆∆CT_ method and normalized to the expression level of GAPDH (glyceraldehyde-3-phosphate dehydrogenase).

### 2.5. Western Blot

RAW 264.7 cells were seeded in a 6-well plate, pretreated with 4-hydroxycinnamaldehyde (12.5 µM and 25 µM) and ECC (200 µg/mL) for 2 h, and then treated with LPS at 100 ng/mL. The cells were lysed in RIPA buffer (50 mM Tris-HCl pH 7.4, 150 mM NaCl, 0.25% deoxycholate, 1% NP-40, and 1 mM EDTA), and the protein concentration was determined using BCA reagent (Thermo Fischer Scientific, Rockford, IL, USA) according to the manufacturer’s instructions. Proteins were separated by sodium dodecyl sulfate polyacrylamide gel electrophoresis (SDS-PAGE) and transferred to polyvinylidene difluoride (PVDF) membranes (Merck Millipore, Darmstadt, Germany). After the transfer, the membranes were blocked with 5% nonfat dried milk/TBST (20 mM Tris pH 7.4, 150 mM NaCl, and 0.2% Tween-20) for 1 h and then incubated overnight at 4 °C with a primary antibody from Cell Signaling Technology (Danvers, MA, USA). After washing with TBST, the membranes were probed with horseradish peroxidase-labeled secondary antibodies (Cell Signaling Technology, Danvers, MA, USA). Finally, the proteins were visualized using enhanced chemiluminescence detection reagent (Thermo Fisher Scientific, Pittsburgh, PA, USA). The densities were normalized to the expression of GAPDH or the total amount of each protein.

### 2.6. Immunofluorescence Staining

RAW 264.7 cells were seeded in an 8-well plate, pretreated with 4-hydroxycinnamaldehyde (12.5 µM and 25 µM) and ECC (200 µg/mL) for 2 h, and then treated with LPS (100 ng/mL) for 22 h. The cells were fixed with 4% paraformaldehyde in PBS for 10 min and blocked with 5% normal goat serum (R&D Systems, Minneapolis, MN, USA) containing 0.3% Triton X-100 in PBS for 1 h. Macrophage cells were probed with primary antibodies overnight at 4 °C. After washing with PBS, the cells were incubated with Alexa Fluor 488-conjugated secondary antibody for 1 h and then counterstained with DAPI (Cell Signaling Technology). Fluorescence images were acquired using an LX51 fluorescence microscope (Olympus, Tokyo, Japan).

### 2.7. Measurement of Intracellular Reactive Oxygen Species (ROS)

RAW 264.7 cells (5 × 10^4^ cells/well) were plated in a 96-well black clear bottom plate (Corning Inc., Corning, NY, USA). After incubation, the cells were treated with 4-hydroxycinnamaldehyde (12.5 µM and 25 µM) and ECC (200 µg/mL) for 2 h and stimulated with LPS (100 ng/mL) for 22 h. The cells were exposed to 20 µM DCFH-DA in PBS, washed twice, and then incubated in the dark for 30 min at 37 °C. They were washed with cold PBS, and the fluorescence intensity was measured at an excitation (485 nm) and emission (535 nm) wavelength using a microplate reader (SPARK 10M; Tecan, Männedorf, Switzerland).

### 2.8. Enzyme-Linked Immunosorbent Assay (ELISA)

RAW 264.7 cells were treated with 4-hydroxycinnamaldehyde (12.5 µM and 25 µM) and ECC (200 µg/mL) for 2 h and stimulated with LPS (100 ng/mL) for 22 h. The concentrations of TNF, IL-6, and IL-1β in the cell supernatants were analyzed using an ELISA kit (R&D Systems) according to the manufacturer’s protocols. Absorbance was determined at 450 nm using a microplate reader.

### 2.9. Animal Experiment Design

Forty male SD rats were purchased from KOATECH (Gyeonggi-do, Korea). All animals were divided into four groups (*n* = 10 per group): control, indomethacin-induced acute stomach injury, positive control (AE), and experimental (ECC). All animals were maintained at 22 ± 2 °C with 55 ± 5% humidity and a 12/12 h light/dark cycle. Rats were fasted for 48 h, and then AE and ECC (10 mL/kg) were orally injected into animals in each group. The dose concentration was determined by referring to previous studies [1]. After 30 min, indomethacin (80 mg/kg) was orally administered to induce acute stomach injury. Five hours later, all animals were sacrificed under inhalation anesthesia with isoflurane, and their stomachs and serum were extracted for experiments. All diets and water were freely provided. This study was approved by the KNOTUS Institutional Animal Care and Use Committee (AEC-20120608-0001; Korea).

### 2.10. Western Blotting of Stomach Tissue

Stomach tissues from the SD rats were homogenized and subjected to Western blot analysis. Tissue proteins were also incubated with primary antibodies against p-p65, p-65, IKKα, IKKb, COX-1, COX-2, and iNOS. The next day, the membrane was washed three times, incubated with the secondary antibody for 1 h, and washed three times. Membranes were reacted with Super Signal West Femto Maximum Sensitivity Substrate (Thermo Fisher Scientific, Pittsburgh, PA, USA) and detected using ChemiDoc (Bio-Rad, Hercules, CA, USA).

### 2.11. Determination of Stomach Injury

The stomachs of all animals were stretched to determine the degree of injury. The damaged area (ulcer index) was analyzed using Image J software (Bethesda, MD, USA) after the images were taken with a digital camera. Ulcer index (%) = (damaged area/whole area of stomach) × 100.

### 2.12. Measurement of Cytokines

Blood was collected from the abdominal vena cava and mixed with clot activator. The blood was clotted for 15 min at RT and then centrifuged at 5000 rpm for 5 min. Serum was harvested, and ELISA was conducted using the manufacturer’s guide books. Prostaglandin E2 (PGE2) and myeloperoxidase (MPO) were analyzed with the harvested supernatant in HGF cell cultures or blood serum from SD rats. PGE2 (Enzo Life Sciences, Farmingdale, NY, USA) and MPO (Thermo Fisher Scientific, Waltham, MA, USA) were detected using ELISA kits.

### 2.13. Statistical Analysis

Analyses were performed using the GraphPad software (GraphPad Prism 5, San Diego, CA, USA). The data were analyzed using one-way analysis of variance to determine differences between the treatment and control groups or the treatment and LPS groups. All data are expressed as the mean ± the standard error of the mean. Values of * *p* < 0.05 were statistically significant.

## 3. Results

### 3.1. Profiling of ECC

**Figure 1:**
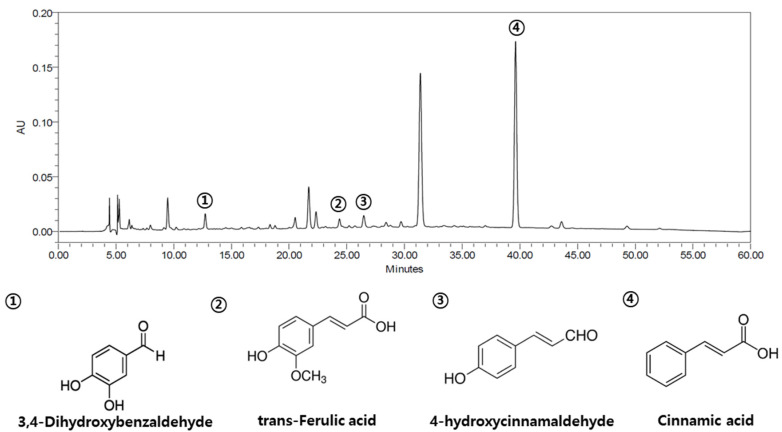
HPLC chromatogram of ECC. ECC was analyzed using Alliance HPLC (Waters e2695, MA, USA) and was used for cell and animal tests. Standardized extract of *Cinnamomum cassia* (ECC).

We performed HPLC analysis to confirm the components of ECC (Figure 1). Four compounds were found to be significant: 4-hydroxycinnamaldehyde, 3-4-dihydroxybenzaldehyde, trans-ferulic acid, and cinnamic acid.

### 3.2. Effect of ECC and Its Components on Cell Viability in RAW 264.7 Cells

**Figure 2:**
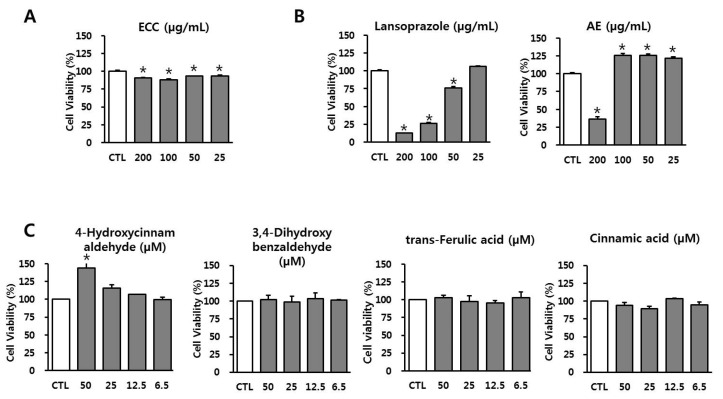
Effect of *Cinnamomum cassia* extract and its components on the viability of RAW 264.7 cells. (**A**) Effect of ECC on cell viability. (**B**) Effects of lansoprazole and *Artemisia* extract (AE) on cell viability. (**C**) Effects of ECC components on cell viability. RAW 264.7 cells were treated with different concentrations of ECC and its components for 24 h. Cell viability was determined using an EZ-Cytox assay as described in the Materials and Methods. The data are expressed as the means ± SD (*n* = 3). * *p* < 0.05 compared to the control group.

We first tested the cellular toxicity of various concentrations (200, 100, 50, and 25 µg/mL, or 50, 25, 12.5, and 6.5 µM, respectively) of ECC and its components in RAW 264.7 cells. As shown in Figure 2, ECC presented no significant cytotoxicity, even at 200 µg/mL (91.05% ± 0.98% and 106% ± 1.63%, respectively). In addition, 4-hydroxycinnamaldehyde, 3-4-dihydroxybenzaldehyde, trans-ferulic acid, and cinnamic acid did not affect the viability of RAW 264.7 cells up to 50 µM. Lansoprazole showed dose-dependent cytotoxicity, and AE was not cytotoxic, except at 200 µg/mL. These data indicate that a high dose (200 µg/mL) of ECC could be used for subsequent experiments.

### 3.3. Anti-Inflammatory Effect of ECC and ECC Components on NO Production in LPS-Stimulated RAW 264.7 Cells

Nitric oxide (NO) is a free radical generated by macrophages as inducible NO during the initial steps of inflammation progression. NO is a major mediator of inflammation [12]. Cellular activation by the cell wall components of Gram-negative bacteria results in the production of many inflammatory mediators in the culture medium [13].

**Figure 3:**
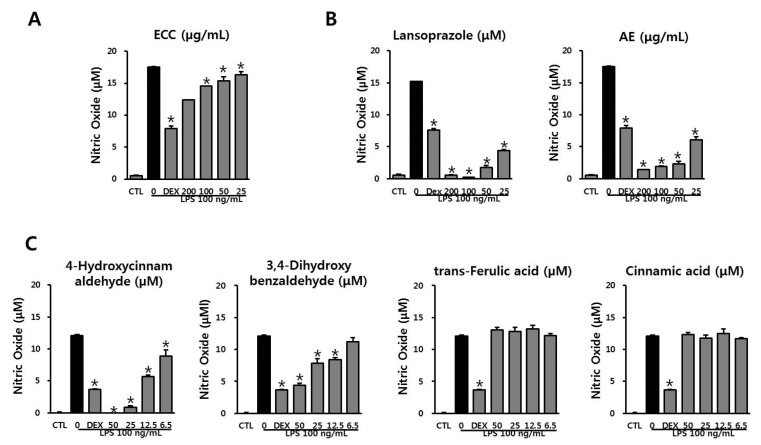
Anti-inflammatory effect of ECC and ECC components on NO production of RAW 264.7 macrophages. (**A**) Effect of ECC on NO production. (**B**) Effects of lansoprazole and *Artemisia* extract (AE) on NO production. (**C**) Effects of ECC components on NO production. RAW 264.7 cells were preincubated with ECC at indicated concentrations (200, 100, 50, and 25 µg/mL, or 50, 25, 12.5, and 6.5 µM) for 2 h, followed LPS (100 ng/mL) for 22 h. NO production was determined using Griess reagent. The data are presented as the means ± SD (*n* = 3). * *p* < 0.05 compared to the LPS group.

We measured NO production in the culture medium to evaluate the anti-inflammatory effects of ECC. LPS treatment stimulated substantial NO production in the cell medium (Figure 3). Dexamethasone, a positive control, decreased NO levels compared to those in the LPS group. 4-Hydroxycinnamaldehyde and 3-4-dihydroxybenzaldehyde dramatically suppressed NO production in a dose-dependent manner. ECC inhibited NO production in a dose-dependent manner by decreasing cell viability. Lansoprazole and AE observed a considerable decrease in NO. However, the inhibition of NO by lansoprazole and AE (200 µg/mL) partly affected the decrease in cell viability (Figure 2). Trans-ferulic and cinnamic acids had no effect on NO production in LPS-induced RAW 264.7 macrophages. Based on the cell viability and nitrite assay data, 200 µg/mL ECC was chosen for further studies.

### 3.4. Anti-Inflammatory Gene Expression of ECC and 4-Hydroxycinnamaldehyde

**Figure 4:**
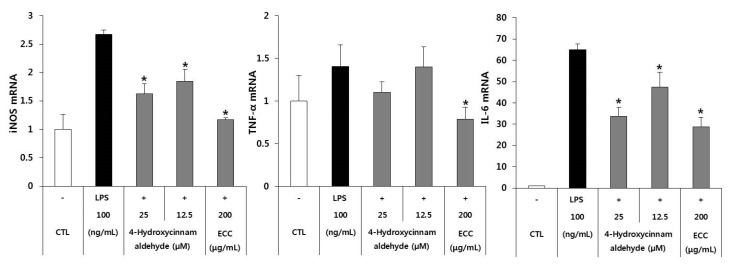
Gene expression of cytokines in RAW 264.7 cells preincubated with ECC or 4-hydroxycinnamaldehyde. The cells were treated with ECC or 4-hydroxycinnamaldehyde for 2 h and then LPS at 100 ng/mL. The relative gene expression was normalized to that of GAPDH expression as an internal control. The data are the means ± SD of three separate experiments. * *p* < 0.05 compared to the LPS group.

Total RNA was isolated using a commercial kit, and mRNA levels of iNOS, TNF-α, and IL-6 were measured by real-time RT-PCR (Figure 4) to determine the effect of ECC and 4-hydroxycinnamaldehyde on the transcription of inflammatory mediators. LPS-treated cells strongly upregulated the expression of iNOS, TNF-α, and IL-6 mRNA compared with the untreated cells. Conversely, the expression of iNOS, TNF-α, and IL-6 was downregulated by the ECC and 4-hydroxycinnamaldehyde treatments. In addition, 4-hydroxycinnamaldehyde suppressed the mRNA expression of iNOS, TNF-α, and IL-6 mRNA in a concentration-dependent manner. These results indicate that both ECC and 4-hydroxycinnamaldehyde were associated with the inhibition of iNOS and TNF-α, and especially IL-6, mRNA expression in RAW 264.7 cells.

### 3.5. Effect of Pro-Inflammatory Cytokins of ECC and 4-Hydroxycinnamaldehyde

**Figure 5:**
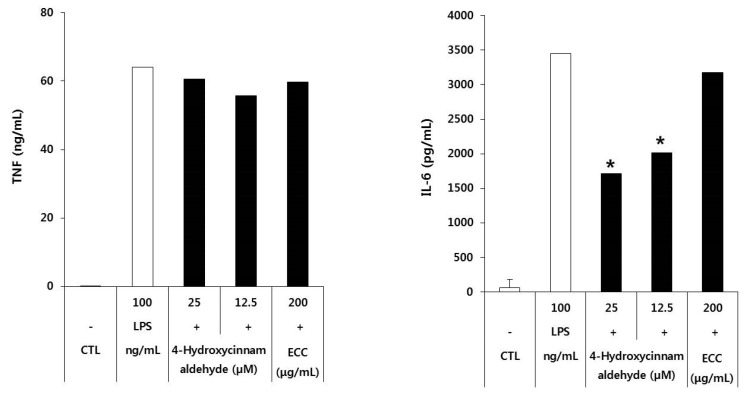
Inhibitory effect of ECC and 4-hydroxycinnamaldehyde on the production of cytokines TNF and IL-6. TNF and IL-6 in the culture supernatant were detected using ELISA. The data are presented as the means ± SD of three independent experiments. * *p* < 0.05 compared to the LPS group.

To examine the inhibitory effect of ECC and 4-hydroxycinnamaldehyde on cytokines in LPS-stimulated RAW 264.7 cells, the cells were pretreated with ECC or 4-hydroxycinnamaldehyde at the indicated doses to measure the secretion of TNF and IL-6 in the supernatant of LPS-treated macrophages. Unlike the gene expression of TNF, secretion of TNF in ECC- and 4-hydroxycinnamaldehyde-treated cells was slightly inhibited compared with that of IL-6 (Figure 5). However, in the case of IL-6, 4-hydroxycinnamaldehyde showed concentration-dependent inhibition, and ECC was weakly reduced when compared with 4-hydroxycinnamaldehyde. The secretion of cytokines (TNF and IL-6) elevated by LPS was reduced by ECC and 4-hydroxycinnamaldehyde.

### 3.6. Effect of ECC and 4-Hydroxycinnamaldehyde on ROS Production and COX-2 and iNOS Protein Expression in LPS-Induced RAW 264.7 Cells

**Figure 6:**
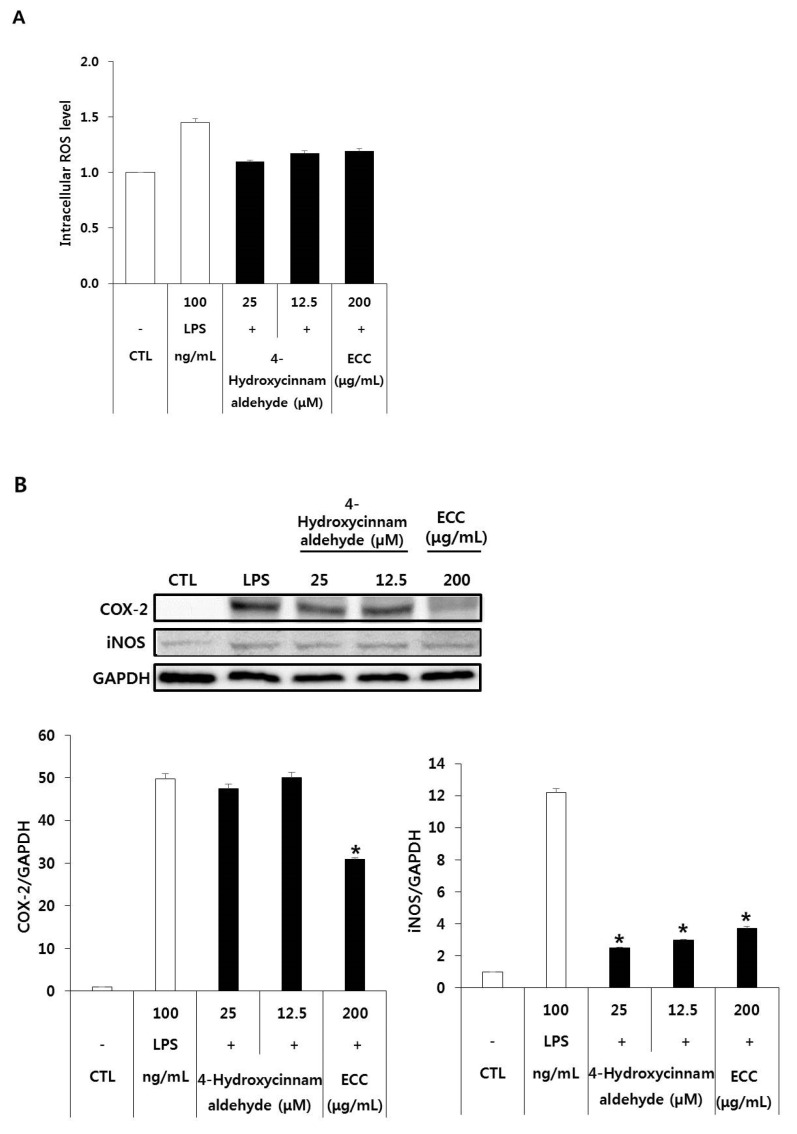
Expression of antioxidant properties and anti-inflammatory mediators of ECC and 4-hydroxycinnamaldehyde in LPS-induced RAW 264.7 cells. The cells were pretreated with ECC or 4-hydroxycinnamaldehyde for 2 h and then stimulated with LPS. (**A**) Intracellular ROS uptake assessed after incubation with 20 µM DCFDA in the dark at 37 °C for 30 min. The fluorescence intensity was measured using a microplate fluorometer. (**B**) COX-2 and iNOS expression induced by ECC and 4-hydroxycinnamaldehyde using Western blotting. The bands of the proteins were densitometrically analyzed by Image J. The data presented are the means ± SD (*n* = 3). * *p* < 0.05 compared to the LPS group.

ROS are byproducts of normal oxygen metabolism and play essential roles in cell signaling and homeostasis [14]. Nonetheless, the overexpression of ROS can cause inflammation and tissue or cellular damage [15]. We examined the accumulation of intracellular ROS and the levels of iNOS and COX-2, inflammatory mediators, induced by ECC and 4-hydroxycinnamaldehyde in LPS-stimulated RAW 264.7 cells. As shown in Figure 6A, ROS accumulation was slightly decreased by ECC and 4-hydroxycinnamaldehyde compared to that in the LPS group. Both ECC and 4-hydroxycinnamaldehyde markedly reduced iNOS and COX-2 protein levels (Figure 6B). In addition, there was more significant inhibition of iNOS expression than COX-2 expression. The results acquired for iNOS protein levels are consistent with those acquired for its mRNA level. Overall, our data indicate that ROS generation decreased due to the inhibition of iNOS and COX-2 expression levels by ECC and 4-hydroxycinnamaldehyde after LPS treatment.

### 3.7. Effect of ECC and 4-Hydroxycinnamaldehyde on the MAPK Pathway in RAW 264.7 Cells

MAPKs play a vital roles in intracellular signaling cascades via which diverse extracellular stimuli converge to launch inflammatory responses. The MAPK pathway is critical for many biological processes, including proliferation, differentiation, apoptosis, inflammation, and responses to environmental stresses [16].

**Figure 7:**
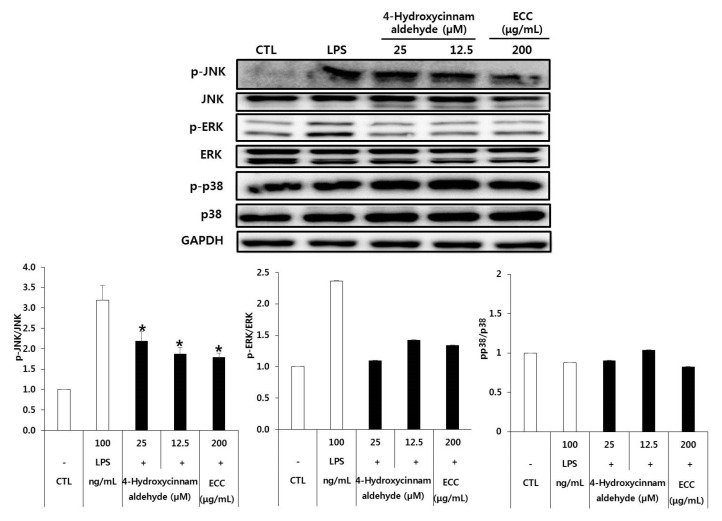
Effect of ECC and 4-hydroxycinnamaldehyde on LPS-activated phosphorylation of the MAPK pathway in macrophages. RAW 264.7 cells were treated with samples for 2 h, followed by treatment with LPS for 22 h. Total cellular extracts were examined for the indicated antibodies using Western blot analysis. Quantification of p-JNK, p-ERK, and p-p38 was normalized to JNK, ERK, and p38 using image J. The data are the means ± SD of at least three separate experiments. * *p* < 0.05 compared to the LPS group.

Therefore, we tested whether MAPKs, including JNK, ERK, and p38, were involved in LPS-induced macrophages after ECC or 4-hydroxycinnamaldehyde treatment. As shown in Figure 7, ECC and 4-hydroxycinnamaldehyde significantly attenuated LPS-stimulated phosphorylation of JNK and ERK but did not inhibit the phosphorylation of p38. In addition, the total expression of ERK, p38, and JNK did not change in murine macrophages treated with ECC or 4-hydroxycinnamaldehyde followed by LPS. Our findings indicate that ECC and 4-hydroxycinnamaldehyde had a crucial effect on LPS-induced phosphorylation of JNK and ERK in RAW264.7 cells by LPS induction.

### 3.8. Regulation of Transcription Factors by ECC or 4-Hydroxycinnamaldehyde in RAW 264.7 Cells

**Figure 8:**
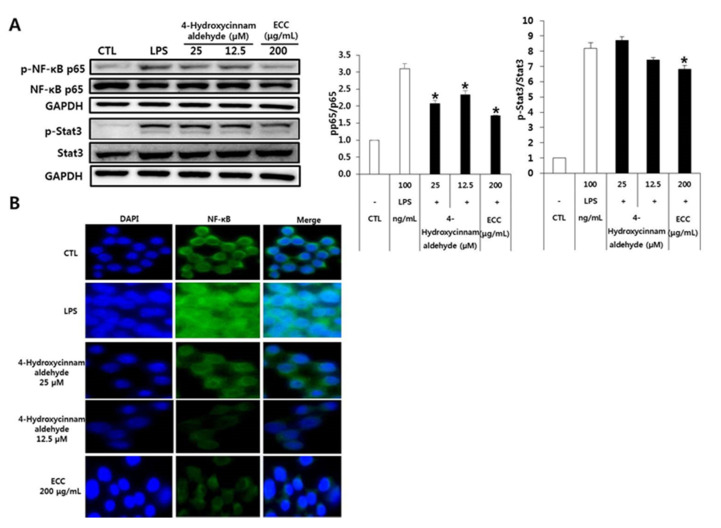
Effect of ECC on the phosphorylation levels of transcription factors in LPS-induced RAW 264.7 cells. (**A**) Effect of ECC on the phosphorylation level of transcription factors using Western blot. Protein extracts were analyzed by Western blot, and quantification of the phosphorylation of NF-κB and STAT proteins was normalized to GAPDH. GAPDH was used as a loading control. Relative expression of each band was analyzed using Image J software. Values represent the mean ± SD (*n* = 3). * *p* < 0.05 compared to the LPS group. (**B**) Effect of ECC on the translocation of NF-κB using a microscope. LPS-stimulated RAW 264.7 cells for nuclear translocation were stained with NF-κB antibody (Alexa Fluor) and counterstained with DAPI. They were observed using a fluorescence microscope (magnification × 60), merged Alexa fluor (NF-κB), and blue (DAPI) images.

NF-κB is an inducible transcription factor that plays a central role in the immune response, inflammatory responses, cellular differentiation, and survival of normal and malignant cells [17]. The transactivation domains of NF-κB are restricted to RelA (p65), RelB, and c-Rel. Among NF-κB dimers, the p65/p50 heterodimer is detected in most cell types and acts as a functional transcription factor. The Janus kinase-signal transducer and activator of transcription (JAK-STAT) cascade has also been reported to be a pivotal inflammatory signaling pathway that could mediate inflammatory disorders [18]. After activation of STAT, STATs translocate into the nucleus and bind to pro-inflammatory mediators. We examined both the NF-κB and STAT3 transcription factors that are mediated by inflammation. As shown in Figure 8, LPS-induced RAW 264.7 cells activated NF-κB p65. However, phosphorylation of p65 was inhibited by ECC and 4-hydroxycinnamaldehyde. Furthermore, STAT3 was markedly phosphorylated in macrophages stimulated with LPS. In contrast, 4-hydroxycinnamaldehyde and ECC attenuated the STAT3 activation. To confirm our Western blotting results, we visualized NF-κB translocation using immunostaining. Only the LPS treatment translocated NF-κB into the nucleus, whereas 4-hydroxycinnamaldehyde and ECC were localized in the cytoplasm.

### 3.9. Ameliorating Effect of ECC on Acute Stomach Injury in Rats

**Figure 9:**
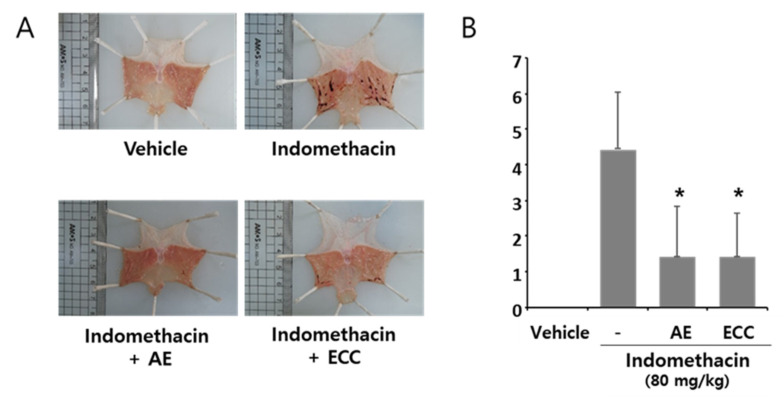
Ameliorating effect of ECC on gastric damage in rats. Forty rats were fasted for 48 h before the experiment. Experimental groups were treated with ECC (24 mg/kg) or AE (20 mg/kg) for 30 min and then with indomethacin (80 mg/kg). All the treatments were administrated orally. Five hours later, all animals were sacrificed, and their stomachs were harvested to measure the damaged areas. (**A**) Representative pictures of stomachs from each group. (**B**) Statistical analysis of the damaged areas. The data represent from values from 10 repeated experiments. * *p* < 0.05 compared to the indomethacin group.

We conducted an animal experiment using ECC in an NSAID-induced acute stomach injury rat model. To measure the damaged area in the stomach, each stretched stomach was analyzed using Image J software, as shown in Figure 9A. We confirmed that indomethacin induced hemorrhage in the stomach. Bleeding was significantly reduced by the ECC or AE treatment (Figure 9B). These data suggest that ECC reduced the damaged area induced by indomethacin.

### 3.10. ECC Ameliorates Acute Stomach Injury by Decreasing Inflammatory Signaling

**Figure 10:**
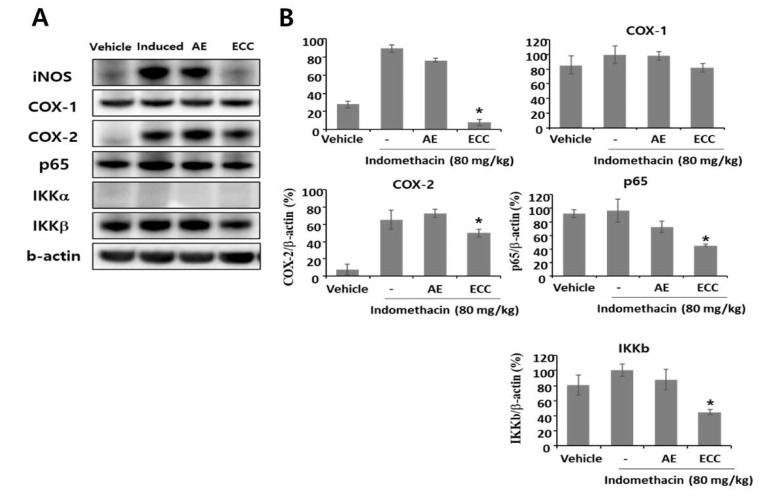
Effects of ECC on inflammatory protein expressions in rat stomachs. Forty rats were fasted for 48 h before the experiment. Experimental groups were treated with *Cinnamomum cassia* extract (CE, 24 mg/kg) or *Artemisia* extract (AE, 20 mg/kg) for 30 min and then with indomethacin (80 mg/kg). All treatments were administrated orally. Five hours later, all animals were sacrificed, and their stomachs were harvested to measure the inflammatory regulators using Western blotting. (**A**) Representative pictures of Western blotting. (**B**) Statistical result. The data represent values from 3 repeated experiments. * *p* < 0.05 compared to the indomethacin group.

To determine whether ECC was able to modulate cyclooxygenase and inflammatory signaling, we performed Western blotting with animal stomachs and ELISA with animal serum. Interestingly, ECC significantly reduced the expression levels of iNOS and COX-2, whereas COX-1 expression did not change in the indomethacin-induced acute stomach (Figure 10). ECC also reduced p-65 and IKKb expression under significant conditions compared to AE expression. Moreover, PGE2 and MPO levels in the serum were also significantly decreased by ECC treatment (Table 1). The PGE2 and MPO levels of the induced group were 843.7 ± 256.9 and 637.9 ± 167.7, which were significantly decreased by ECC to 434.3 ± 62.1 and 181.8 ± 90.0, respectively. This suggests that ECC was able to reduce specific COX-2 expression and inflammatory signaling molecule levels, including PGE2, p-65, and IKKb.

## 4. Discussion

NO is produced by NOS and has been shown to have a number of significant biological functions, including tumor cell killing and host defense against intracellular pathogens. iNOS is not present when the cells are inactive but is induced by microbial components such as LPS [19]. Therefore, many studies have been conducted to effectively regulate NO in the immune response using LPS-induced RAW 264.7 macrophages.

*C. cassia* has a variety of pharmacological activities, including anti-inflammatory, antidiabetic, anti-obesity, antitumor, antibacterial, antiviral, and immunoregulatory effects [9,20]. Traditional herbal medicines have been used for centuries, despite the scientific insufficiency of their therapeutic efficacies, and therefore a large number of studies have been conducted on the effectiveness of extracts or compounds. *C. cassia* extract, or compounds derived from *C. cassia*, confirmed the effects of inflammation induced by LPS in the gastritis model [21]. Moreover, few studies have reported on *C. cassia* in both in vitro and in vivo studies. The pharmacological mitigation of LPS-inducible inflammatory mediators, such as NO and TNF-α, is considered one of the conditions to relieve the immune response caused by the activation of macrophages. Therefore, RAW 264.7 macrophages provide us with a great model for anti-inflammatory drug screening [10].

The primary objective of the present study was to demonstrate the anti-inflammatory effects and the underlying mechanism of ECC in LPS-induced RAW 264.7 cells and NSAID-induced gastritis in rats. First, we evaluated the cellular toxicity of various concentrations of ECC and ECC components for 24 h in murine macrophages. Consequentially, there was little toxicity in all tested samples except for lansoprazole and the 200 µg/mL concentration of AE. Hong et al. [9] also reported that cinnamon water extract (CWE) concentrations up to 400 μg/mL were not cytotoxic in peritoneal macrophages treated with LPS or not.

4-Hydroxycinnamaldehyde and 3, 4-dihydroxybenzaldehyde dramatically attenuated NO production in LPS-stimulated RAW 264.7 cells. In addition, ECC decreased NO levels in a dose-dependent manner. The level of NO in lansoprazole and AE showed much stronger suppression than that in dexamethasone, which was used as a positive control.

Kwon [20] demonstrated that cytokine-induced NO production in RINm5F cells was completely blocked at a cortex cinnamomi extract concentration of 1.0 mg/mL. However, we examined the gene expression of iNOS, TNF-α, and IL-6 with 200 μg/mL of ECC compared to 4-hydroxycinnamaldehyde. The 4-hydroxycinnamaldehyde treatment inhibited the expression of iNOS, TNF-α, and IL-6 in a dose-dependent manner in LPS-induced RAW 264.7 cells. Surprisingly, compared to 4-hydroxycinnamaldehyde, ECC strongly downregulated the mRNA levels of iNOS, IL-6, and TNF-α. However, the levels of the cytokines TNF-α and IL-6 in cell-free culture supernatants were different from their gene expression levels. 4-Hydroxycinnamaldehyde potently suppressed the cytokine IL-6 in a concentration-dependent manner. According to a study by Lv et al. [22], TNF mRNA levels gradually increased in a time–dependent manner after 20 h of exposure in LPS-treated murine macrophages, but TNF cytokine release peaked at 8 h. The data showed that the increase in TNF mRNA expression does not occur simultaneously with TNF release.

ROS production from intracellular antioxidant reactions leads to oxidative stress and is associated with various pathological conditions. The levels of inflammatory mediators and cytokines are increased in macrophages, which, in turn, accelerate ROS accumulation in the cells. The ability of ECC and 4-hydroxycinnamaldehyde to reduce ROS formation was evaluated using DCFDA, an intracellular ROS-sensitive dye. ROS accumulation in LPS stimulated the macrophages. Pretreatment with ECC or 4-hydroxycinnamaldehyde decreased LPS-induced ROS production in RAW 264.7 cells induced by LPS. ROS serve as secondary messengers and regulate inflammatory signaling pathways [23,24]. To investigate the influence of inflammatory mediators such as iNOS and COX-2, Western blotting was performed. The protein expression of iNOS and COX-2 was inhibited by ECC and 4-hydroxycinnamaldehyde in LPS-activated macrophages. These data corroborate the idea that ECC and 4-hydroxycinnamaldehyde possess antioxidant and anti-inflammatory effects. The theoretical possibility for NO confirmed that the change in NO release was influenced by the iNOS content [25].

Many studies have reported that activation of the MAPK signaling pathway plays an important role in TLR4-activated macrophages and anti-inflammatory mechanisms [26,27]. Our results reveal that the phosphorylation of JNK and ERK was downregulated by ECC preincubation in LPS-stimulated RAW 264.7 cells, and it was also inhibited by 4-hydroxycinnamaldehyde, but not dose-dependently in the case of JNK, whereas LPS did not stimulate the activation of p38. The RAW cell line does not express MKP5 (MAPK phosphatase, also known as Dusp10), which negatively regulates MAPKs. MKP5 expression was induced as early as 15 min after LPS exposure. Therefore, activation of JNK, but not p38, was observed in MKP5-deficient macrophages following LPS treatment [7]. We infer that this is due to changes in the LPS-induced inflammatory mediator at each time point.

This study showed that the LPS-induced expression of iNOS and COX-2 is regulated through the MAPK signaling pathways. ECC plays an important role in the downregulation of ERK and JNK signaling. Signaling of TLR4 in LPS-stimulated macrophages induces the activation of MAPKs, PI3K/Akt, and the IB kinase complex, subsequently leading to the activation of NF-κB [28,29]. Therefore, we investigated whether ECC and 4-hydroxycinnamaldehyde in LPS-induced macrophages are regulated by transcription factors, such as NF-κB and STAT3. We confirmed that phosphorylation of NF-κB and STAT3 was downregulated by the ECC and 4-hydroxycinnamaldehyde treatments in murine macrophages, followed by LPS exposure. However, NF-κB showed more potent regulation than STAT3. In addition, we showed the effects of ECC and 4-hydroxycinnamaldehyde on LPS-induced NF-κB translocation into the nucleus. The results indicate that both ECC and 4-hydroxycinnamalde in the presence of LPS sequestered in the cytoplasm. These combined findings suggest that LPS-stimulated NF-κB and STAT3 are inactivated by ECC or 4-hydroxycinnamaldehyde, resulting in the inhibition of pro-inflammatory mediators due to the accumulation of NF-κB in the cytoplasm.

In our animal study, ECC strongly inhibited p65, IKKa, IKKb, and COX-2 expression. The mechanism of NSAID-induced gastritis is impaired mucosal defense by decreased prostaglandin and inhibition of epithelial cell proliferation by suppressing epidermal growth factor, which eventually causes ulceration and bleeding in the stomach [30]. The signaling pathways involved in NSAID-induced gastritis are apoptosis and NF-κB inflammation [31,32]. High doses of NSAIDs, including indomethacin, ibuprofen, and sulindac, generally induce apoptosis [33,34].

**Figure 11:**
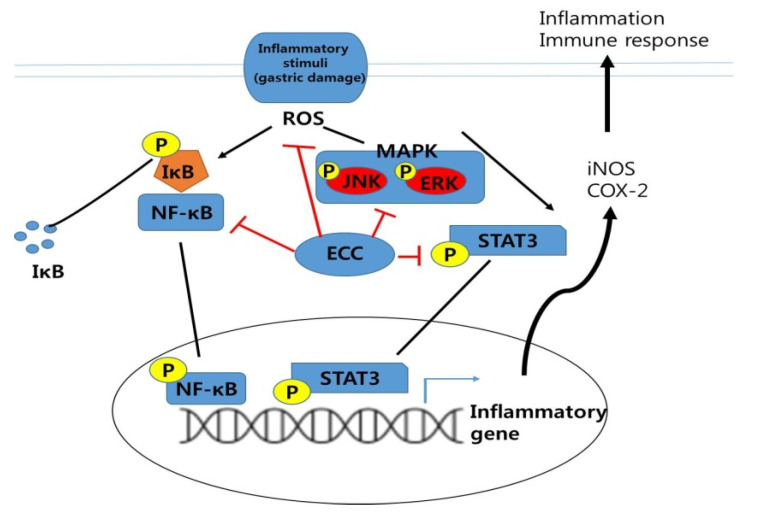
Schematic illustration of the mechanism of ECC against inflammation (gastric damage).

The reduced expression levels of p65, IKKa, and IKKb by ECC resulted in diminished gastritis. Importantly, we demonstrated the efficacy of ECC in an acute gastritis model and found that the damaged area of the stomach was ameliorated by ECC because ECC might have a precise inhibitory effect on p65 and IKKb expression but not on IKKa. We also confirmed that PGE2 levels in the serum were significantly reduced, which might be due to the reduced COX-2 expression by ECC. p65 is a critical transcriptional factor in inflammation after translocation into the nucleus as inflammation is triggered [35]. Previous reports revealed that p65, other than IKKa and IKKb, is considered to be a major regulator of NF-κB signaling based on knockout studies [36,37,38,39]. It is very important that ECC affected p65 rather than IKKb and IKKa in vitro and in vivo (Figure 11), demonstrating that ECC could aid in the recovery from bleeding in indomethacin-induced acute gastritis.

## 5. Conclusions

In conclusion, *C. cassia* and 4-hydroxycinnamaldehyde exhibited anti-inflammatory and antioxidant effects in murine macrophages. They significantly decreased LPS-induced iNOS, TNF, and IL-6 gene expression and secretion in RAW 264.7 cells. ECC, a natural product, has antioxidant properties that can reduce intracellular ROS production. The ability of ECC and 4-hydroxycinnamaldehyde to inhibit inflammation and oxidative damage is mediated through the inhibition of the MAPK, NF-κB, and STAT3 signaling pathways. We also confirmed that ECC exerted an effect on indomethacin-induced gastritis. Thus, ECC has the potential to serve as an alternative therapeutic agent for gastritis.

## Figures and Tables

**Table 1:** Effects of ECC on serum PGE2 and MPO.

	PGE_2_	MPO
Vehicle	225.5 ± 60.6	34.1 ± 19.8
Indomethacin	843.7 ± 256.9	637.9 ± 167.7
Indomethacin + AE	434.3 ± 121.9 *	203.4 ± 61.2 *
Indomethacin + ECC	434.6 ± 62.1 *	181.8 ± 90.0 *

Forty rats were fasted for 48 h before the experiment. The experimental groups were treated with ECC (10 mL/kg) or AE (10 mL/kg) for 30 min, followed by indomethacin (80 mg/kg). Five hours later, all the treatments were administered orally. Then, all animals were sacrificed, and their blood was harvested to measure PGE2 and MPO levels. * *p* < 0.05 compared to the indomethacin group.

## Data Availability

Data is contained within the article.
